# Unveiling Variations in Electronic and Atomic Structures Due to Nanoscale Wurtzite and Zinc Blende Phase Separation in GaAs Nanowires

**DOI:** 10.1021/acs.nanolett.4c01262

**Published:** 2024-05-20

**Authors:** Lunjie Zeng, Eva Olsson

**Affiliations:** Department of Physics, Chalmers University of Technology, SE-41296 Gothenburg, Sweden

**Keywords:** phase separation, III−V nanowires, band gap, band alignment, atomic structure, electron energy loss spectroscopy

## Abstract

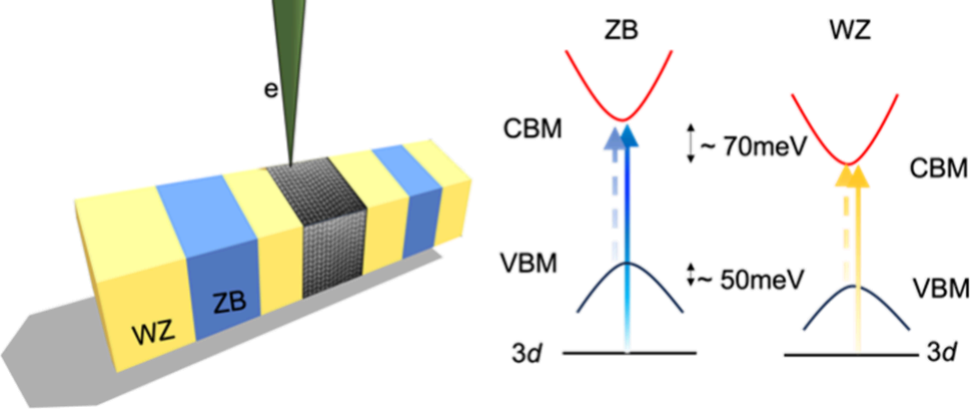

Phase separation is an intriguing phenomenon often found in III–V nanostructures, but its effect on the atomic and electronic structures of III–V nanomaterials is still not fully understood. Here we study the variations in atomic arrangement and band structure due to the coexistence of wurtzite (WZ) and zinc blende (ZB) phases in single GaAs nanowires by using scanning transmission electron microscopy and monochromated electron energy loss spectroscopy. The WZ lattice distances are found to be larger (by ∼1%), along both the nanowire length direction and the perpendicular direction, than the ZB lattice. The band gap of the WZ phase is ∼20 meV smaller than that of the ZB phase. A shift of ∼70 meV in the conduction band edge between the two phases is also found. The direct and local measurements in single GaAs nanowires reveal important effects of phase separation on the properties of individual III–V nanostructures.

Structure polytypism in III–V nanostructures has attracted extensive research interest because it provides an ideal platform for understanding phase separation as well as for exploring novel electronic devices at the nano and atomic scales. In bulk form, III–V compound semiconductors most often exist in the zinc blende (ZB) phase.^1^ But when grown with nanoscale dimensions, many III–V materials could form crystals with the energetically less favorable wurtzite (WZ) phase.^2−8^ ZB and WZ phases normally coexist in III–V nanowires due to the competition of the two phases in a vapor–liquid–solid (VLS) growth process.^2,7,9−14^ The WZ lattice has a different symmetry and unit cell structure compared to the ZB phase, which would result in differences in electronic band structure and electronic and optical properties.^15^ The structure phase competition during growth causes atomically sharp interfaces between the polytypes, thus providing an effective way to engineer electronic structure and fabricate atomically sharp heterojunctions in single nanowire devices.^16−21^

GaAs nanowires are some of the most studied III–V nanowires due to their outstanding electronic and optoelectronic properties.^22−26^ Structure phase separation has been found in GaAs nanowires.^13,17,27^ Consequently, GaAs nanowires have often been used as a prototype for understanding the effect of structure polytype and phase separation on the electronic band structures, as well as electronic and optical properties of III–V nanostructures.^17,28−35^ In spite of extensive experimental and theoretical investigations, a quantitative understanding of the effect of phase separation on the electronic structure of GaAs nanowires is still under debate.^29,30,35−38^ For example, there is a wide span in the reported band gap energies of the WZ GaAs phase.^31,36,38−40^ There is even no consensus as to whether the WZ band gap is smaller or larger than the ZB band gap.^30^ There is also a considerably large discrepancy in the reported results with regard to band offsets of the valence bands and conduction bands between the two phases.^29,31,41,42^ The main challenge in reliable measurements of the electronic structure of the ZB and WZ phases in single nanowires is due to the limited spatial resolution of the techniques used and the small dimensions of the nanowires and phase-separated domains. The commonly used optical techniques, such as photoluminescence and Raman spectroscopy, as well as cathodoluminescence, often have a spatial resolution larger than the dimensions (diameters) of the nanowires and the nanoscale phase-separated domains.^32,33,35−38,42^ Furthermore, simultaneous high-resolution crystal and electronic structure analyses that could directly correlate the local electronic structure with lattice structure information is still lacking. As a result, there is an urgent need for an experimental investigation that can simultaneously provide crystal structure and electronic structure information from individual ZB and WZ domains at high spatial resolution.

In this study, we used aberration-corrected scanning transmission electron microscopy (STEM) together with monochromated electron energy loss spectroscopy (EELS) to investigate the effect of phase separation on the crystal and electronic structures of GaAs nanowires with coexisting ZB and WZ domains. ZB and WZ domains in a single GaAs nanowire were clearly identified, and subtle differences in atomic structure between the two phases were found. Monochromated STEM-EELS was used to locally measure interband transitions and core level excitations in the ZB and WZ phases, revealing differences in the electronic structures of the two phases. The joint density of states (JDOS) between the top of the valence band and the bottom of the conduction band shows significant differences between the two phases. The Ga 3d core-level excitations unveil a shift of the conduction band minimum. The band offsets between the two phases are also discussed based on the EELS measurements.

Individual GaAs nanowires consist of nanoscale ZB and WZ domains along the axial direction. The length of the nanowires is usually 10–15 μm. The bottom region of the nanowire contains only the ZB structure (Figure 1a). A small segment at the tip of the nanowire, with a length of around 1 μm, shows predominantly a WZ structure (Figure 1a). Between the two regions, there is an intermixture of nanoscale ZB and WZ domains. The contrast variation along the nanowire shown in the TEM bright field (BF) image (Figure 1a) originates from a change in lattice orientation between the adjacent domains. Domain width along the axial direction varies between less than 1 nm and a few hundred nm. The ZB and WZ structures have very similar atomic arrangements, though the ZB phase has a face-centered-cubic (fcc) lattice structure and the WZ phase has a hexagonal-close-packed (hcp) unit cell. The lattice structure difference between the ZB and WZ phases can be best described by the difference in the stacking order of close-packed GaAs lattice planes along the ZB [111] (WZ [0001]) direction (Figure 1b–d). The ZB phase shows an ABCABC··· stacking sequence, while the WZ phase has an ABAB··· type of stacking (Figure 1b,d). Atomic resolution STEM annular dark field (ADF) images clearly show the lattice structures of the pure ZB phase, ZB/WZ mixing, and the pure WZ phase in the nanowire (Figure 1b–d). The same chemical composition and the close correlation between the atomic arrangements in the ZB and WZ phases in III–V nanowires have often led to the assumption that the WZ phase has an ideal hcp unit cell and that the lattice constants of the two phases follow simple geometric relationships.^29,31,39^ Under this assumption, the lattice distances of the close-packed planes and the unit cell volumes are the same in the two phases.

**Figure 1:**
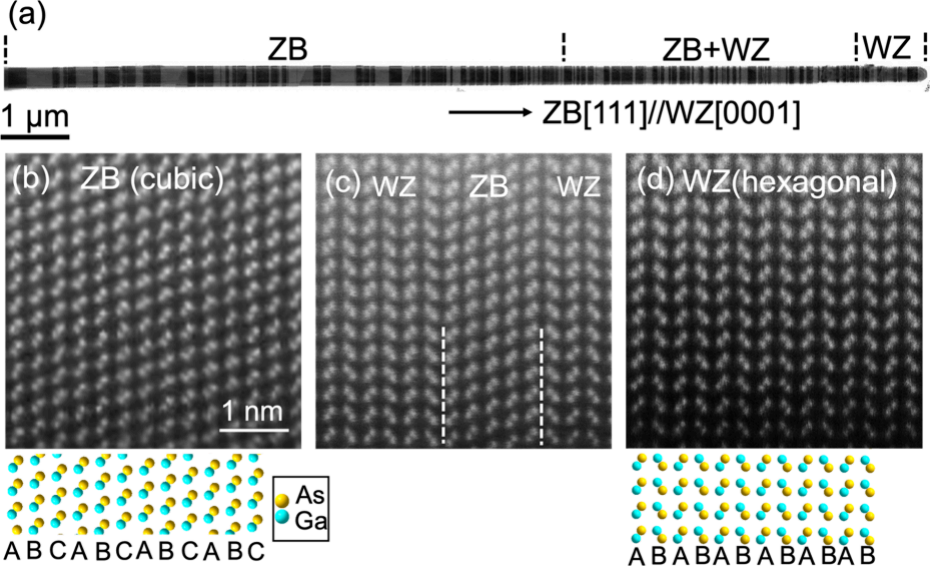
Structure of a GaAs nanowire with a mixture of zinc blende (ZB) and wurtzite (WZ) phases viewed along the ZB [1–10] (equivalent to WZ [1–100]) zone axis. (a) Low-magnification TEM bright field (BF) image of the whole nanowire. The ZB-dominated region is close to the bottom (growth substrate) of the nanowire, while the WZ region is near the nanowire tip. In between, there is a region where nanoscale ZB and WZ domains are intermixed. (b) Atomic resolution STEM ADF image of the ZB lattice in the ZB region. A schematic of the ABCABC··· stacking sequence of the GaAs atomic planes in ZB GaAs is shown below the image. (c) Atomic resolution STEM ADF image of a region with nanoscale ZB/WZ switching. The dashed lines indicate the boundaries between ZB and WZ domains. (d) Atomic resolution STEM ADF image of a WZ domain. The ABAB··· stacking sequence of the GaAs atomic planes in the WZ phase is shown below the image.

Contrary to the assumption, subtle differences in the lattice structure between the two phases were unveiled by atomic resolution STEM imaging. The lattice distances between the close-packed planes in the ZB and WZ crystals were measured directly based on the atomic resolution STEM ADF images (Figure 2a). STEM image intensity line profiles along the ZB [111] (WZ [0001]) direction were compared (Figure 2b). The peaks in the line profiles show the positions of the close-packed lattice planes, each of which consists of a Ga and an As atomic plane. By aligning the first peak to the left in the profiles, the shift in peak position is clearly visible (Figure 2b). The peaks of the WZ structure are shifted to the right compared to those of the ZB lattice, showing that the lattice plane distance in the WZ phase is around 1.5% larger than that in the ZB phase. The lattice plane spacings are about 3.39 ± 0.02 and 3.44 ± 0.02 Å for the ZB phase and WZ phases, respectively. Similar measurements also reveal that lattice spacing in the ZB domains along the ZB [22–4] (WZ [10–10]) direction is smaller than that in the WZ phase (see section S1 in the Supporting Information). The fast Fourier transform (FFT) patterns of the atomic resolution STEM images were used to further examine the differences in lattice spacing along the nanowire axial direction and the perpendicular direction. Line profiles across the central spots of the FFT patterns and along the two directions are compared (Figure 2c,d). When aligning the central peaks in the profiles, the high-order spots from the two phases show a small but visible mismatch. For example, the WZ (0008) peak is closer to the central spot than the ZB (444) peak, indicating a larger lattice distance along the nanowire axial direction in the WZ phase, consistent with that measured using real space images (Figure 2b). Similarly, in the line profiles along the direction perpendicular to the nanowire length direction, a subtle mismatch between the WZ (30–30) peak and ZB (22–4) peak is found. It shows that the WZ lattice spacing along the perpendicular direction is also larger than that in ZB, by ∼1%. According to the assumption that the WZ phase has an ideal hcp unit cell, WZ (30–30) and ZB (22–4) should have the same lattice spacing. Thus, WZ lattice constants along both the nanowire axial and the perpendicular directions are larger than those obtained via simple transformation of the ZB unit cell.

**Figure 2:**
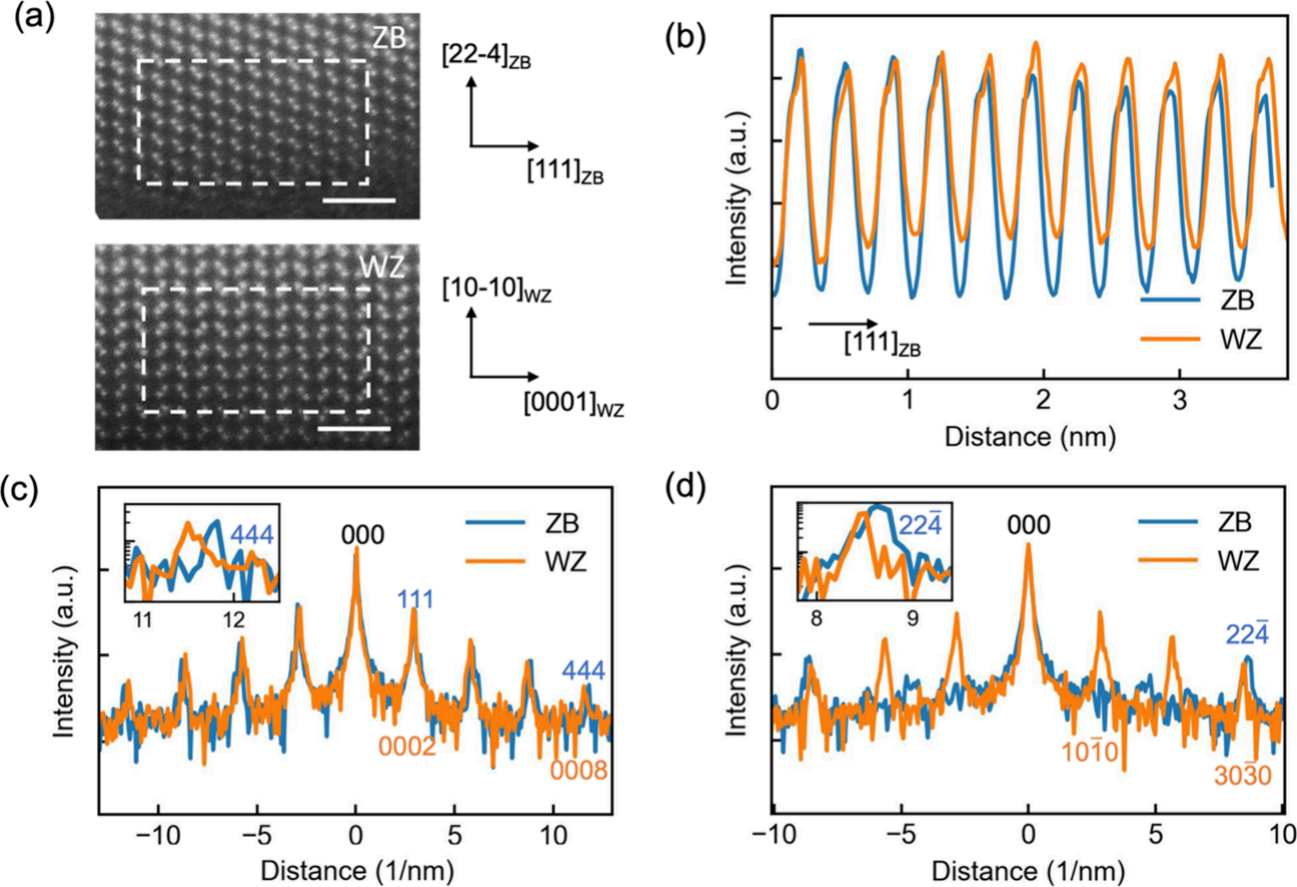
Differences in atomic structure between the ZB and WZ phases in a single GaAs nanowire. (a) Atomic resolution STEM ADF images of ZB (top) and WZ (bottom) used for the structure analysis. The scale bars are 1 nm. As shown in Figure 1, the images were acquired with the electron beam incident along the ZB [1–10] direction. Line profiles were extracted from the areas indicated by the dashed windows. (b) Line profiles of the STEM image signal from the dashed windows marked in (a). The image intensity profiles are plotted along the ZB [111] direction, showing the differences in lattice distances between the close-packed planes, i.e. ZB (111) and WZ (0002) lattice planes. The STEM signal within each window was summed up along the perpendicular direction (the ZB [22–4] direction). (c,d) Comparison of line profiles of fast Fourier transform (FFT) patterns obtained from atomic resolution STEM images of the ZB and WZ phases. In (c), line profiles along the 111_ZB_ (0002_WZ_) direction are plotted. In (d), line profiles along the 22–4_ZB_ (10–10_WZ_) direction are shown. Miller indexes of a few of the peaks are labeled for ZB (blue) and WZ (orange). Each inset in (c) and (d) shows an enlarged area of the corresponding line profile, more clearly demonstrating the shift in peak positions in the FFT patterns.

The electronic band structures of the ZB and WZ phases within a single nanowire also show different characteristics. The band gaps of the two phases were analyzed by using valence EELS (VEELS) measurements. The measurements were carried out at the middle of ZB and WZ domains with axial dimensions of about 200 nm (see section S2 in the Supporting Information), minimizing surface contribution and signal mixing between the different structural phases due to the delocalization effect on VEELS signals.^43,44^ It is known that surface and retardation effects can modify the EELS signal at the band gap onset in dielectric materials.^45−47^ For reliable measurements and comparison of the two phases, sample and experimental conditions were carefully considered and kept the same for the measurements on the two different phases (Methods and sections S2 and S3 in the Supporting Information). Spectrum onsets of the energy loss signal in both spectra appear around 1.4 eV. The onsets in VEELS signal are often used to characterize the band gaps of semiconductors and insulators.^45,48−50^ There is a small but clear shift in the band gap onset between the two phases (Figure 3a). The band gap onset of the VEELS of the ZB phase is determined to be 1.42 (±0.005) eV, which is close to the well-documented band gap energy of ZB GaAs in both bulk form and nanostructures at room temperature.^36,38,51,52^ The band gap onset of the WZ phase is about 20 meV smaller than that of the ZB phase (see section S4 in the Supporting Information for additional data).

**Figure 3:**
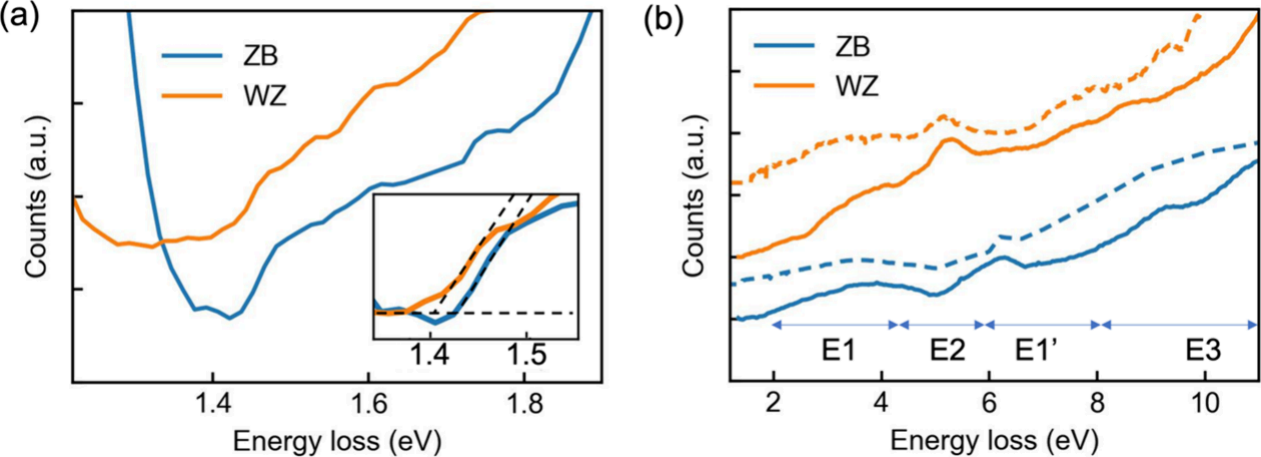
VEELS from the ZB and WZ phases in the GaAs nanowire, showing fine structures from interband transitions. The spectra are shifted vertically for clarity. (a) Spectra in the energy range between ∼1.2 and ∼1.9 eV showing band gap onsets of the two phases around 1.4 eV. Inset: a magnified view of the spectra around the band gap onset of ∼1.4 eV. The baseline signal intensity of the spectra before the band gap onset is indicated by the horizontal dashed line. The linear fit to the EELS signal above the onset is shown by the inclined dashed lines. (b) Spectra in the energy range from ∼2 to ∼11 eV showing fine structures related to interband transitions above band gap excitation. The energy range is divided into four segments, E1, E2, E1′, and E3, for facilitating discussions of the origins of the signals. Solid lines are experimental data, while dashed lines are simulated spectra.

The effect of phase separation on the electronic band structure close to the Fermi level and, in turn, the optical properties of GaAs nanowires are evident in VEELS fine structures above band gap onset (Figure 3b). These spectrum features are mainly ascribed to essential interband transitions from the states close to the valence band top to the states close to the conduction band bottom, or joint density of states (JDOS). JDOS combines the energy dispersion of the valence and conduction bands involved in the transitions, determining the optical dielectric function. Simulation of EELS spectra was carried out by taking the materials’ optical property (dielectric function), retardation, and surface effects into account (see section S3 in the Supporting Information).^46,53^ The simulated spectrum agrees well with the experimental data for the ZB phase in terms of the positions of the fine features (Figure 3b). The agreement holds even in the energy loss range between ∼2 and 5 eV, where retardation and surface effects strongly influence the EELS signal and change the shape of the spectrum. The implication of the high-degree agreement between simulation and experiment for the ZB phase is 2-fold. First, the bulk dielectric function of ZB GaAs can be used to describe the optical properties of the ZB phase in the nanowire.^54^ Second, the theoretical model that considers bulk effect, surface, and retardation effects is suitable to model VEELS in nanowire structures. Following previous discussions on the dielectric functions of several III–V semiconductors, including GaAs, we divide the EELS spectrum in the energy range from ∼2 to ∼11 eV into 4 regions, E1 (between 2 and ∼4.5 eV), E2 (between ∼4.5 and 6 eV), E1′ (between 6 and 8 eV), and E3 (8 and 11 eV),^55−58^ as shown in Figure 3b. The interband transition signal (modified by surface and retardation effects) in segment E1 is believed to mainly come from transitions from the highest spin–orbit-split valence bands to the lowest conduction band at L point and along the **Λ** line in the Brillouin zone (BZ)^57,58^ (see section S5 in the Supporting Information for band diagram of GaAs). The transitions in E2 are assigned to a region of parallel bands in the **ΓX**UL plane in BZ, causing strong oscillation in the EELS signal near 5 eV.^57,58^ The transitions in E1′ are ascribed to those from the highest valence bands to the second lowest conduction band in the vicinity of L point in the BZ.^57,58^ We note that signals in E3 have not been reported before and may be due to transitions that involve a lower valence band and the third lowest conduction bands around the X point, which have larger bandwidth and less distinct structure in DOS in contrast to bands closer to the Fermi level.

The experimental EELS spectrum of the WZ phase shows distinctively different fine structures compared to the VEELS spectrum of the ZB phase (Figure 3b), indicating the difference in dielectric function and electronic structure caused by phase separation. The WZ phase has lower symmetry than the ZB structure, though the two phases have largely similar atomic arrangement in terms of the distance and number of first, second, and third nearest neighbors. The band structure change in WZ has been predicted previously through theoretical calculations using various techniques.^29,31,34,39,41^ The dielectric function of WZ GaAs has also been calculated based on a theoretical band structure model.^59^ The modeled dielectric function was used to simulate the VEELS of the WZ phase in the nanowire. In terms of line shape, the simulated WZ spectrum coincides well with the experimental data in the spectrum range from ∼2 to ∼7 eV (Figure 3b). The largest discrepancy between simulation and experiment exists in the signal above 7 eV, where both signals show two minor peaks between 7 and 10 eV, but the peaks are shifted to slightly lower energy in the simulation with respect to the experimental data.

The similarities between the experimental EELS spectrum and the simulation for the WZ phase indicate that the pseudopotential calculation in the previous theoretical study^39^ largely captures the main features in WZ electronic band structure close to the Fermi level. However, as in many previous studies, the band structure calculation overlooked the lattice structure difference between the ZB and the WZ phases. The differences in lattice distances (Figure 2) suggest changes in bond lengths, which inevitably affect the ionicity of the chemical bonding, as well as the band structure. The observed discrepancy between the VEELS experiment and simulation for the interband transitions that involve high-energy level conduction bands may be due to the small changes in lattice parameters in GaAs nanowires.

The conduction band DOS characteristics of the ZB and WZ phases in the nanowires were further investigated by core-loss EELS. Figure 4a shows the Ga-M_4,5_ EELS edges of the ZB and WZ phases in the nanowire with edge onsets at around 20 eV. Because of the flatness of the core levels, the energy loss near edge fine structure (ELNES) in the spectra is essentially determined by the DOS close to the bottom of the conduction bands.^60−63^ The Ga-M_4,5_ onset in the WZ phase is shifted slightly to lower energy, by ∼70 meV, compared with that in the ZB lattice (Figure 4a). Apart from the relative shift of the spectra, the ELNES from the two phases shows similar general features but with subtle differences. In ZB Ga-M_4,5_ ELNES, there are two main peaks with peak positions at ∼21.8 and ∼23.7 eV, respectively. There is also a minor peak between 24.5 and 25.5 eV. The first main peak at around 21.8 eV is predominantly due to the excitations from 3d levels to the two lowest conduction band minima near X and L, where the bands show pronounced p character.^56,62,64^ The second peak around 23.7 eV in ZB ELNES is believed to originate from excitations from 3d levels to the second lowest conduction bands at L. The minor peak between 24.5 and 25.5 eV is likely from the excitations to the third lowest conduction band, with low scattering cross-section. In WZ Ga-M_4,5_ ELNES, the first main peak shows a line shape and width very similar to those in the ZB spectrum. However, the WZ spectrum shows two minor peaks after the first main peak. The changes in ELNES unravel the conduction band structure differences between the two phases: the WZ conduction band edge is shifted (∼70 meV) toward lower energy compared to the ZB conduction bands as evidenced by the shift in EELS edge onsets and the main ELNES features. The lowest WZ conduction band seems to have a DOS similar to that of ZB, indicated by the very similar first main peak in the spectra. The two phases show more distinctively different structures in DOS for the second and third lowest conduction bands.

**Figure 4:**
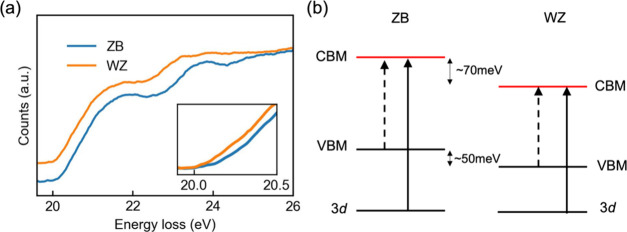
Ga 3d core-level excitation EELS signals and band offsets of ZB and WZ phases in the nanowire. (a) Background subtracted Ga 3d core-loss spectra (see section S6 in the Supporting Information for background subtraction). The spectra are shifted vertically for clarity. Inset: magnified view of the edge onset in the spectra. The baseline signals of the two spectra before the edge onsets are aligned for visualizing the relative shift in energy of the edges. (b) Schematic of band offsets between the conduction band minimum (CBM) and valence band maximum (VBM) in the ZB and WZ phases in the nanowire. The dashed arrow indicates the excitation corresponding to the band gap onset in VEELS. The solid arrow shows the 3d core-level excitation that gives rise to the Ga 3d core-loss EELS signal.

Furthermore, the combination of VEELS and core-loss EELS measurements reveals band offsets at the band edges between ZB and WZ phases (Figure 4b). VEELS shows that the band gap of the WZ phase is smaller than the ZB crystal, by ∼20 meV, while core-loss measurement suggests a downshift of the conduction band minima of ∼70 meV for the WZ phase. Consequently, the valence band top in the WZ phase should be about 50 meV lower than that of the ZB phase. Such a band offset is consistent with previous optical spectroscopy measurements and will affect the band alignment at ZB/WZ junctions, which are commonly found in GaAs nanowires grown through the VLS process.^42^

In summary, the crystal structure and electronic structure of ZB and WZ phases in single GaAs nanowires were studied and compared using atomic resolution STEM imaging and monochromated STEM-EELS. The results show that the WZ lattice has a larger lattice spacing along both the nanowire axial direction and the perpendicular direction, as opposed to the common assumption that the two phases have the same lattice spacings. The band gap of the WZ phase is ∼20 meV smaller than that of the ZB phase. The dielectric function calculation of the WZ phase captures the main features in the energy interval between 2 and 7 eV,^39,59^ with discrepancies between 7 and 11 eV. The conduction band edge of the WZ phase is shifted by ∼70 meV toward lower energy compared to the ZB phase. The conduction band DOS shows similar features for the first and second lowest conduction bands in ZB and WZ domains, but there are considerable differences in DOS for higher level conduction bands in the two phases.

## Supporting Information Available

Methods, lattice spacings along ZB[22–4] direction, ZB and WZ domains, VEELS modeling, band gap onset shift, ZB band diagram, and background subtraction for core-loss spectra (PDF)
