# Supplementary material for: Unveiling Variations in Electronic and Atomic Structures Due to Nanoscale Wurtzite and Zinc Blende Phase Separation in GaAs Nanowires
Source: Nano Lett. 2024 May 20;24(22):6644–50. doi: 10.1021/acs.nanolett.4c01262 (PMC11157649; doi:10.1021/acs.nanolett.4c01262)
Supplement: Supplementary file 1 — nl4c01262_si_001.pdf [file nl4c01262_si_001.pdf]

Supporting Information

Unveiling variations in electronic and atomic structures due to nanoscale Wurtzite and Zinc Blende phase separation in GaAs nanowires

Lunjie Zeng* and Eva Olsson*

Department of Physics, Chalmers University of Technology, SE-41296, Gothenburg, Sweden

Corresponding authors:

Lunjie Zeng: lunjie@chalmers.se

Eva Olsson: eva.olsson@chalmers.se

Supporting Information

Methods

The GaAs nanowires were grown on Si(111) substrates using a molecular beam epitaxy (MBE) system by a self-catalysed vapor-liquid-solid (VLS) method [1].

Individual nanowires were mechanically transferred onto holey carbon film coated Cu TEM grid for TEM experiments. The TEM used was a JEOL monochromated ARM200F equipped with a Schottky field emission gun (FEG), a double-Wien monochromator, a CEOS ASCOR probe C_s corrector, a CEOS CETCOR image C_s corrector, a Continuum Gatan image filter (GIF), as well as a Rio CMOS GIF camera. TEM bright field (BF) images were acquired at 200 kV. For high resolution STEM imaging, the microscope was operated at 200 kV with the monochromator in off mode. The spatial resolution was estimated to be around 1 Å. For EELS measurements, with the monochromator on, a 60 kV accelerating voltage was used. The energy resolution was set to be around 100 meV by selecting a 2μm monochromator slit. The dispersion of the EELS detector was 5 meV/channel. A dual-EELS mode was used for both low-loss and core-loss measurements. During STEM-EELS experiments, the beam convergence half-angle was ~ 26 mrad and the EELS collection half-angle was ~ 10 mrad. To increase the beam current for the enhancement of the signal/noise ratio for EELS, the condenser lens system of the microscope was adjusted. As a result, the beam size became around 2~3 Å during EELS measurements, but the beam size was still small enough for identifying the WZ and ZB phases during monochromated STEM imaging and EELS measurements. EELS measurements were performed on nanowire areas where the nanowire diameter was around 150 nm and on domains that were about 200 nm wide along the axial

direction. The thickness (diameter) of the nanowire is chosen for reliable band gap onset measurement and minimizing surface signal from the top and bottom surfaces of the nanowires (See S3 for effects of thickness on VEELS signals). In addition, the EELS data were acquired at the centre of the domains for further minimizing surface effects from the nanowire side facets and delocalization effect (see S2) on valence EELS signals. Valence and core-loss EELS spectra of each phase were acquired from the same sample locations.

S1 Lattice spacings along ZB[22-4] direction

Image intensity line profiles were also measured to compare lattice spacing of the ZB and WZ phases along the Zinc Blende (ZB) [22-4] direction, using atomic resolution scanning transmission electron microscopy (STEM) annular dark field (ADF) images. The results are shown in Figure S1. As shown in Figure S1(c), the peaks in the WZ line profile are shifted to the right with respect to the ZB peaks, showing slightly larger lattice spacing along the $[22\text{-}4]_{ZB}$ (equivalent to $[10\text{-}10]_{WZ}$) direction in the WZ phase. This result is consistent with the changes in reciprocal space distances shown in Figure 1(d) in the main text.

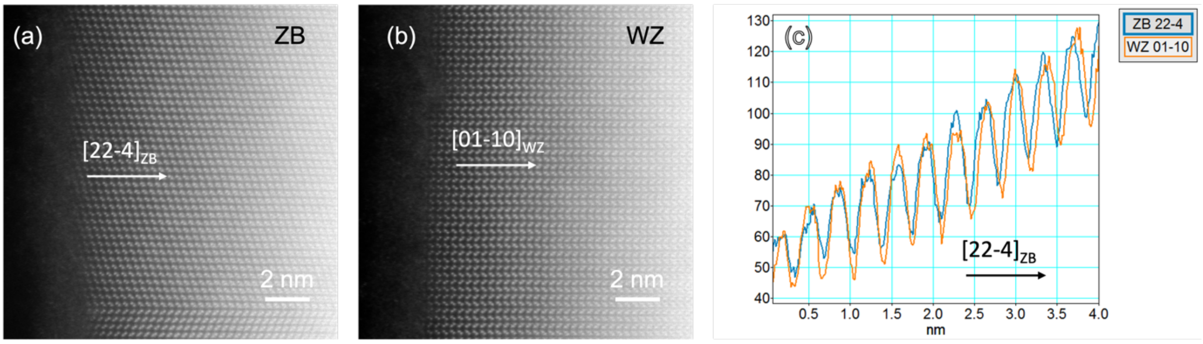

Figure S1. (a)(b) Atomic resolution STEM ADF images of the ZB and WZ phases in a GaAs nanowire, respectively. (c) Comparison of image intensity line profiles obtained from (a) and (b). Line profiles were measured along the $[22\text{-}4]_{ZB}$ (equivalent to $[10\text{-}10]_{WZ}$) direction. The direction is marked in (a) and (b). The line profiles were aligned at the first peak to the left.

S2 ZB and WZ domains

In each nanowire, there are ZB dominated region and WZ-rich region. In the ZB region, there are ZB twin domains. Individual ZB domains with an axial dimension larger than 200 nm can be easily found (Figure S2 (a) – (d)). Some ZB domains are even longer than 1μm. On average, WZ domains are smaller than the ZB domains, but there are WZ domains with an axial size around or larger than 200 nm in each nanowire (Figure S2(e)-(h)). For both ZB and WZ regions, the lattice structure is pure ZB or WZ, except at the twin domain boundaries for the ZB region or stacking faults for the WZ region. Both the twin domains and stacking faults often consist of only two atomic planes along the nanowire axial direction.

Inelastic electron scattering is a delocalized process. Delocalization affects the attainable spatial resolution in EELS measurements and may complicate data interpretation. The degree of the delocalization effect in inelastic electron scattering depends on many factors, including the energy loss signal (the type and energy of the signal), the sample thickness, and the incident beam energy. Previous theoretical and experimental work has already provided quantitative understanding of delocalization in EELS measurements [2–4], though the full picture of the delocalization process is still evolving. In general, EELS signals decrease exponentially as a function of distance to the beam position. Signals at lower energy loss are more delocalized than those at higher energy loss. The signals in the lowest energy range in this work are the interband transition signals at and above the GaAs band gap value (1.4 eV). The low-loss signal in this energy range decreases significantly (~ an order of magnitude) at a distance about 20 to 30 nm away from the beam position, according to previous studies [2–4]. Even EELS

signals at energies as low as ~ 100 meV were shown to reduce about 70% when the beam is about 50 nm away from the sample [5]. We also tested the effect by moving the beam away from the nanowire edge. At about 50 nm distance to the edge, bulk low-loss signal is diminished. Therefore, we anticipate that the signals from regions that are more than 50 nm away from the beam are minimal in our STEM-EELS measurements.

We performed EELS measurements in the ZB and WZ domains about 200 nm long along the nanowire length direction. The beam with a diameter of about 1Å was placed in the middle of the domains, so the signal should solely come from the domain that the beam is located on. Contributions from the adjacent domains as well as domain boundaries should be negligible.

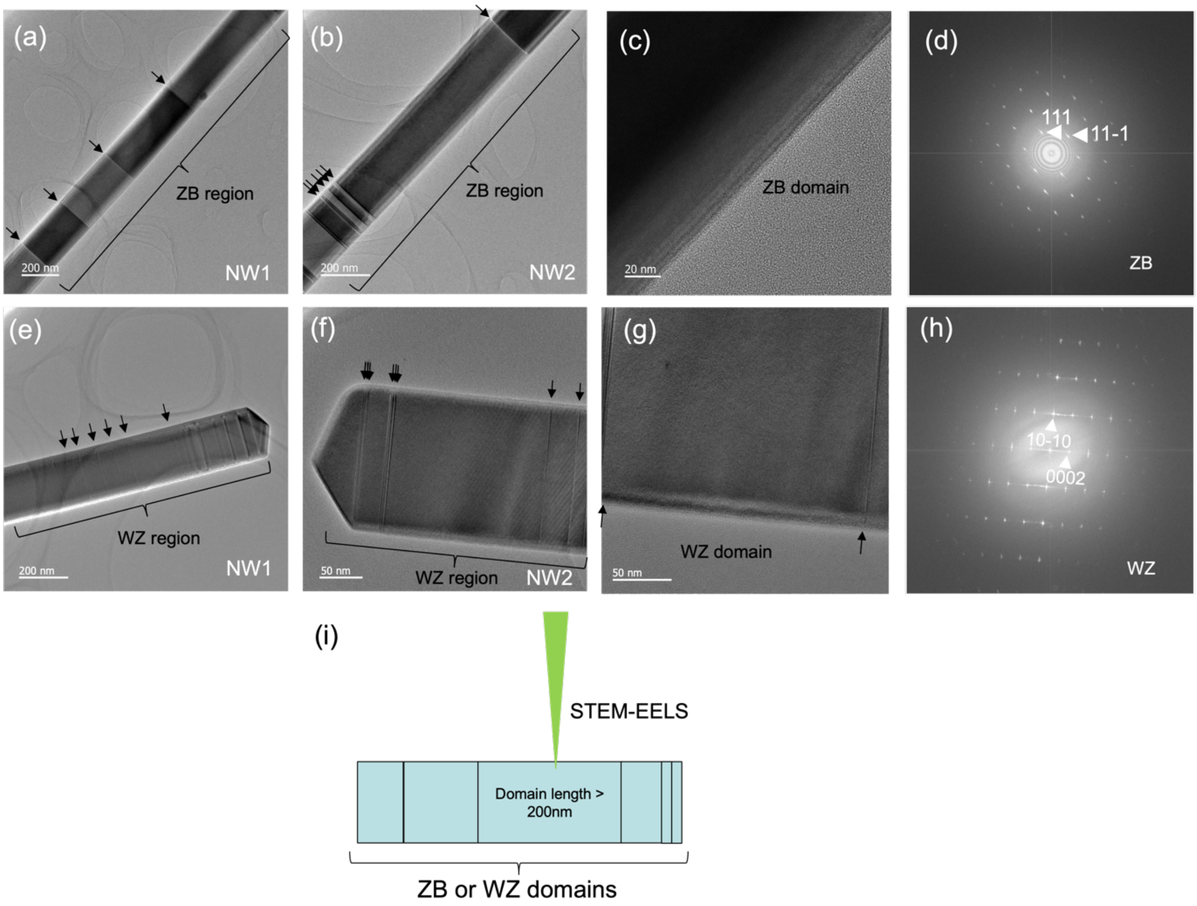

Figure S2. (a)-(c) TEM images of ZB dominated regions. (a) and (b) are images from two different nanowires. Contrast variation (diffraction contrast) between adjacent domains in the images is due to the switching in lattice orientation between the ZB twin domains. Individual ZB domains with axial dimensions larger than 200 nm are visible in both (a) and (b). In (b), the biggest domain is around 1 μm in length. In (c), a single ZB domain with a length larger than 200 nm is shown at atomic resolution. (d) Fast Fourier transform (FFT) pattern of (c), showing the single crystalline structure of the ZB domain. (e)-(g) TEM images of the WZ-dominated regions in the nanowires. Arrows mark the locations of stacking faults in the otherwise WZ structure. (h) FFT pattern of (g) showing the pure WZ structure of the domains. (i) Schematic showing that STEM-EELS was performed at the center of ZB or WZ domains with axial dimensions larger than 200 nm.

Vertical dark lines are domains boundaries or stacking faults in the ZB- or WZ-dominated region.

S3 VEELS modelling

Valence EELS simulation was carried out using the dielectric formulation of inelastic electron scattering in solids [6]. In this formulation, the energy loss signal is essentially governed by the energy loss function (ELF) as well as some experimental parameters, such as sample thickness, beam convergence angle and EELS collection angle. ELF is given by $ELF = \mathrm{Im}[-\frac{1}{\varepsilon(q,\omega)}]$, where $\varepsilon(\mathrm{q}, \omega)$ is the complex dielectric function of the material, with q and ω being wave vector and angular frequency, respectively. Depending on the incoming electron beam energy, the dielectric constants of the materials and the specimen thickness, the retardation and surface effects may also contribute significantly to VEELS, in addition to the intrinsic interband transition signals[7,8]. Based on such a formalism, VEELS simulations were carried out using a Matlab simulation program [6].

For the simulations in this work, the electron beam energy was set to 60 kV and the EELS collection angle was 10 mrad, similar as the experimental values we used. The input dielectric function of bulk Zinc Blende (ZB) GaAs was from a database [9]. Simulations were done for a series of sample thicknesses, from 1 nm to 10 μm.

In dielectric materials, the relativistic effects and retardation effects in electron-matter scattering can be significant and may contribute to energy loss signal in the low-loss region, obscuring band gap measurements [7,8,10]. The theoretical threshold energy for exciting Cerenkov radiation in GaAs is around 25 kV [11], which is much lower than the electron beam energy used in this study. In addition, surface effects may also affect the valence EELS signal close to the band gap onset due to the large surface/bulk ratio in nanowire structures[7,12].

Our simulation results as well as previous study[7] show that, if the sample thickness is ~ 500 nm and above, retardation effects start to shift the band gap onset towards lower energy. When the sample is below 10 nm, surface effect dominates, and consequently the bulk band gap onset signal is weak (Figure S3(a)). In the thickness range between 50 and 250 nm, the band gap onset is not shifted. Based on the simulations, a sample thickness between 100 and 200 nm is suitable for band gap onset detection for GaAs.

Furthermore, when the sample is thin, less than ~ 50 nm thick, surface effect drastically modifies the interband transition fine structure between band gap onset and bulk plasmon in the VEELS signals (Figure S3(b)). Thus, a sample thickness above 50 nm is beneficial for revealing the bulk properties of GaAs nanowires in VEELS. The sample thickness for the nanowires is similar as the nanowire diameter. The diameter of the GaAs nanowires used in this study is in the range between 100 and 200 nm (see Methods in Supporting Information and Figure 1 in the main text).

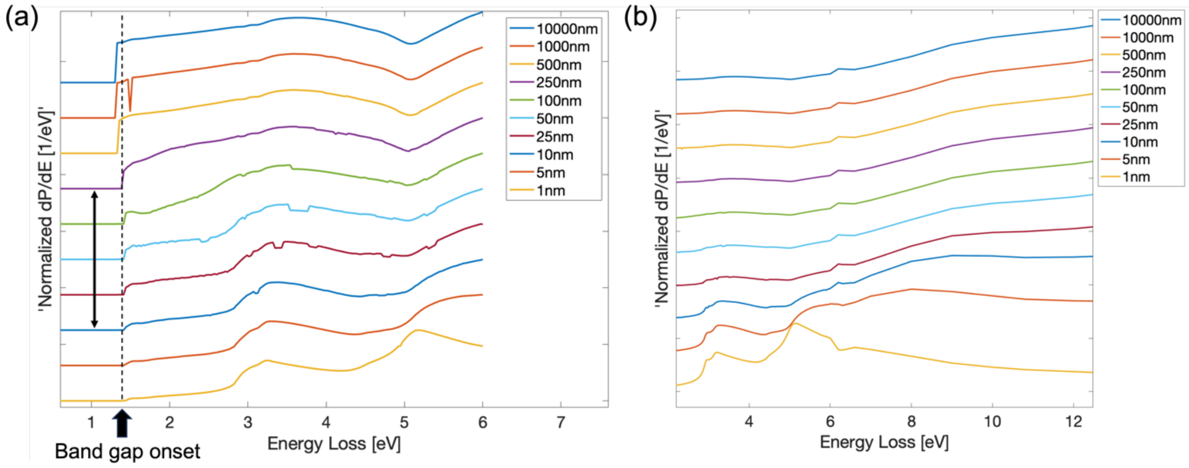

Figure S3. Simulated valence EELS spectra of ZB GaAs at different sample thicknesses in the energy loss ranges of (a) ~0.5 - 6 eV and (b) ~2 - 12 eV. In (a), the dashed line indicates the band gap energy of GaAs at ~ 1.42 eV at room temperature [13]. The double-

arrowed line shows the spectra in the sample thickness range between 10 and 250 nm where the band gap onset doesn't shift and matches the expected band gap energy.

S4 Band gap onset shift

The shift in band gap onset in low-loss EELS spectra of the ZB and WZ phases has been observed in all the nanowires we studied. In total, EELS measurements were performed on three nanowires. In each nanowire, two ZB domains with an axial dimension larger than 200 nm and one WZ domain with a dimension around 200 nm (see S2) were used. Below are spectra from a nanowire different from that shown in the main text (Figure 3). Band gap onsets around 1.4 eV, as well as a slight shift between the onsets, are visible. The onsets were determined by the intersection between the linear fit of the signal directly above the onset and the signal baseline. Similar as the result shown in Figure 3 in the main text, the WZ onset is about 22 meV lower in energy than that of ZB. The uncertainty of onset determination mainly comes from the linear fitting of the signals and is about ±5 meV. As a result, the determined onset shift has an uncertainty of ±10 meV.

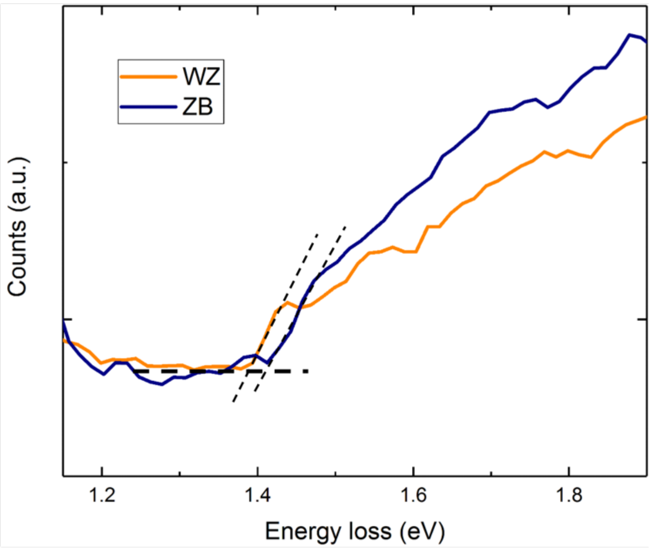

Figure S4. Low-loss EELS spectra of WZ and ZB phases in a different nanowire from that shown in the main text. Band gap onsets were determined by the intersection between

the fitting of the linear part of the EELS signal directly above the onset and the signal baseline. The WZ onset is shifted to lower energy compared to that of ZB. The shift is about 22±10 meV.

S5 ZB band diagram

To facilitate the discussion in the main text, band structure of ZB GaAs was simulated in the energy range between -10eV and 10 eV. The simulation was done using Quantum Espresso [14,15]. We used the plane wave pseudopotential method implemented in Quantum Espresso (PWscf package). Pseudopotentials from standard solid state pseudopotentials (SSSP) database on Materials Cloud were used [16]. The kinetic energy cut-off for wavefunctions was 60 Rydberg, and the convergence threshold for self-consistency was 10^{-6} Rydberg. Electronic structure was calculated using an 8 x 8 x 8 k-mesh. Note that the simulation and the resulted band diagram were intended as an approximate illustration of the band structure, to facilitate the discussions of the VEELS signals in the main text. As a result, we only concern with the main features and band morphology in the band diagram. Other effects, such as spin-orbital coupling, were not considered in the calculations. The band diagram we obtained is consistent with those of ZB GaAs published before [17,18].

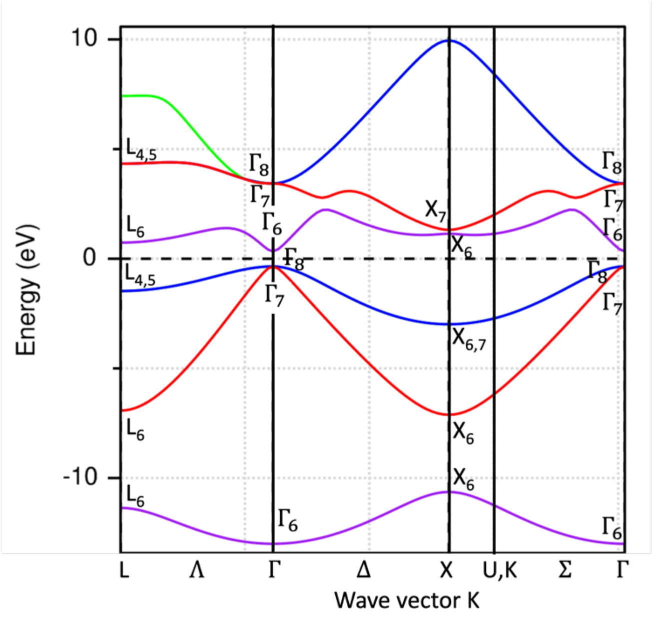

Figure S5. Simulated band diagram of ZB GaAs. 3 highest valence bands and 3 lowest conduction bands are shown.

S6 Background subtraction for core-loss spectra

The original spectra for the WZ and ZB phases that show the Ga-M edges are plotted in Figure S6(a). The shift of the edge onsets and the changes in energy loss near edge structure (ELNES) between the two phases are already visible in the raw data. To better visualize the EELS core-loss signal, the background of the core-loss Ga-M EELS signal was subtracted from raw spectra using the power-law model (Figure S6(b) and (c)), which is commonly used for EELS background subtraction. The background subtracted spectra are shown in Figure 4 in the main text. To make the background subtraction procedure consistent, the same energy window (19 eV – 20 eV) before the edge onsets was used for modeling the background for both the ZB and WZ spectra.

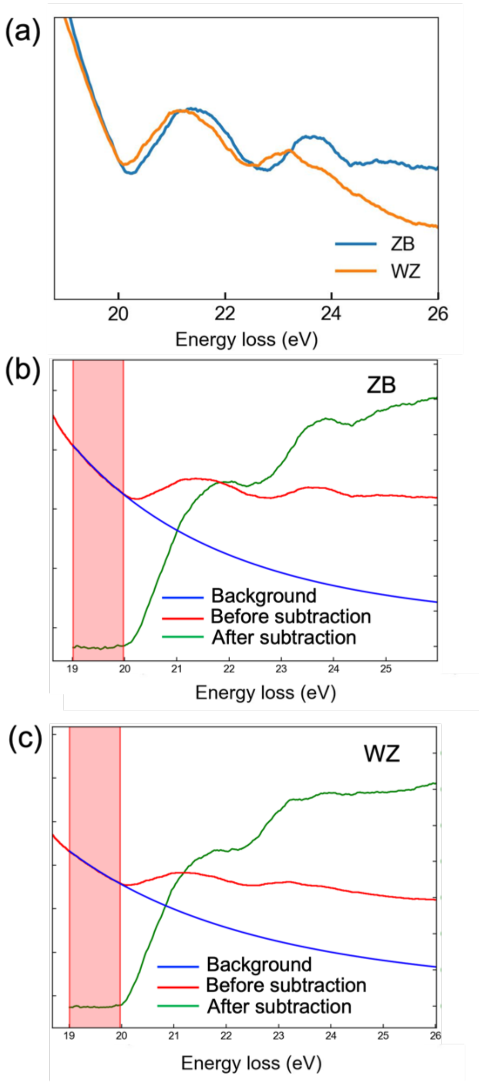

Figure S6. (a) Original EELS spectra of the ZB and the WZ phases in the GaAs nanowire showing the Ga-M edges. (b) and (c) Power-law background subtraction for the ZB and WZ spectra, respectively. The red windows before the EELS edge onsets mark the energy range (19 eV – 20 eV) used for modeling the background signal using the power-law model.

References:

(1) Casadei, A.; Krogstrup, P.; Heiss, M.; Röhr, J. A.; Colombo, C.; Ruelle, T.; Upadhyay, S.; Sørensen, C. B.; Nygård, J.; Fontcuberta i Morral, A. Doping Incorporation Paths in Catalyst-Free Be-Doped GaAs Nanowires. *Appl. Phys. Lett.* **2013**, *102* (1), 013117. https://doi.org/10.1063/1.4772020.

(2) Muller, D. A.; Silcox, J. Delocalization in Inelastic Scattering. *Ultramicroscopy* **1995**, *59* (1–4), 195–213. https://doi.org/10.1016/0304-3991(95)00029-Z.

(3) Egerton, R. F. Limits to the Spatial, Energy and Momentum Resolution of Electron Energy-Loss Spectroscopy. *Ultramicroscopy* **2007**, *107* (8), 575–586. https://doi.org/10.1016/j.ultramic.2006.11.005.

(4) Egerton, R. F. Scattering Delocalization and Radiation Damage in STEM-EELS. *Ultramicroscopy* **2017**, *180*, 115–124. https://doi.org/10.1016/J.ULTRAMIC.2017.02.007.

(5) Krivanek, O. L.; Lovejoy, T. C.; Dellby, N.; Aoki, T.; Carpenter, R. W.; Rez, P.; Soignard, E.; Zhu, J.; Batson, P. E.; Lagos, M. J.; Egerton, R. F.; Crozier, P. A. Vibrational Spectroscopy in the Electron Microscope. *Nature* **2014**, *514* (7521), 209–212. https://doi.org/10.1038/nature13870.

(6) R.F. Egerton. *Electron Energy-Loss Spectroscopy in the Electron Microscope*, 3rd ed.; Springer New York, NY, 2011. https://doi.org/https://doi.org/10.1007/978-1-4419-9583-4.

(7) Erni, R.; Browning, N. D. The Impact of Surface and Retardation Losses on Valence Electron Energy-Loss Spectroscopy. *Ultramicroscopy* **2008**, *108* (2), 84–

99. https://doi.org/10.1016/j.ultramic.2007.03.005.

(8) Stöger-Pollach, M. Optical Properties and Bandgaps from Low Loss EELS: Pitfalls and Solutions. *Micron*. Pergamon December 1, 2008, pp 1092–1110. https://doi.org/10.1016/j.micron.2008.01.023.

(9) Palik, E. *Handbook of Optical Constants of Solids*; 1997. https://doi.org/10.1016/B978-012544415-6.50143-6.

(10) Kröger, E. Transition Radiation, Cerenkov Radiation and Energy Losses of Relativistic Charged Particles Traversing Thin Foils at Oblique Incidence - Theoretical Calculations and Numerical Computations. *Zeitschrift für Phys.* **1970**, *235* (5), 403–421. https://doi.org/10.1007/BF01394931/METRICS.

(11) Horák, M.; Stöger-Pollach, M. The Čerenkov Limit of Si, GaAs and GaP in Electron Energy Loss Spectrometry. *Ultramicroscopy* **2015**, *157*, 73–78. https://doi.org/10.1016/j.ultramic.2015.06.005.

(12) Crozier, P. A. Vibrational and Valence Aloof Beam EELS: A Potential Tool for Nondestructive Characterization of Nanoparticle Surfaces. *Ultramicroscopy* **2017**, *180*, 104–114. https://doi.org/10.1016/J.ULTRAMIC.2017.03.011.

(13) Adachi, S. *GaAs and Related Materials*; WORLD SCIENTIFIC, 1994. https://doi.org/10.1142/2508.

(14) Giannozzi, P.; Andreussi, O.; Brumme, T.; Bunau, O.; Buongiorno Nardelli, M.; Calandra, M.; Car, R.; Cavazzoni, C.; Ceresoli, D.; Cococcioni, M.; Colonna, N.; Carnimeo, I.; Dal Corso, A.; de Gironcoli, S.; Delugas, P.; DiStasio, R. A.; Ferretti, A.; Floris, A.; Fratesi, G.; Fugallo, G.; Gebauer, R.; Gerstmann, U.; Giustino, F.;

Gorni, T.; Jia, J.; Kawamura, M.; Ko, H.-Y.; Kokalj, A.; Küçükbenli, E.; Lazzeri, M.; Marsili, M.; Marzari, N.; Mauri, F.; Nguyen, N. L.; Nguyen, H.-V.; Otero-de-la-Roza, A.; Paulatto, L.; Poncé, S.; Rocca, D.; Sabatini, R.; Santra, B.; Schlipf, M.; Seitsonen, A. P.; Smogunov, A.; Timrov, I.; Thonhauser, T.; Umari, P.; Vast, N.; Wu, X.; Baroni, S. Advanced Capabilities for Materials Modelling with Quantum ESPRESSO. *J. Phys. Condens. Matter* **2017**, *29* (46), 465901. https://doi.org/10.1088/1361-648X/aa8f79.

(15) Giannozzi, P.; Baroni, S.; Bonini, N.; Calandra, M.; Car, R.; Cavazzoni, C.; Ceresoli, D.; Chiarotti, G. L.; Cococcioni, M.; Dabo, I.; Dal Corso, A.; de Gironcoli, S.; Fabris, S.; Fratesi, G.; Gebauer, R.; Gerstmann, U.; Gougoussis, C.; Kokalj, A.; Lazzeri, M.; Martin-Samos, L.; Marzari, N.; Mauri, F.; Mazzarello, R.; Paolini, S.; Pasquarello, A.; Paulatto, L.; Sbraccia, C.; Scandolo, S.; Sclauzero, G.; Seitsonen, A. P.; Smogunov, A.; Umari, P.; Wentzcovitch, R. M. QUANTUM ESPRESSO: A Modular and Open-Source Software Project for Quantum Simulations of Materials. *J. Phys. Condens. Matter* **2009**, *21* (39), 395502. https://doi.org/10.1088/0953-8984/21/39/395502.

(16) Talirz, L.; Kumbhar, S.; Passaro, E.; Yakutovich, A. V.; Granata, V.; Gargiulo, F.; Borelli, M.; Uhrin, M.; Huber, S. P.; Zoupanos, S.; Adorf, C. S.; Andersen, C. W.; Schütt, O.; Pignedoli, C. A.; Passerone, D.; VandeVondele, J.; Schulthess, T. C.; Smit, B.; Pizzi, G.; Marzari, N. Materials Cloud, a Platform for Open Computational Science. *Sci. Data 2020 71* **2020**, *7* (1), 1–12. https://doi.org/10.1038/s41597-020-00637-5.

(17) Chelikowsky, J. R.; Cohen, M. L. Nonlocal Pseudopotential Calculations for the

Electronic Structure of Eleven Diamond and Zinc-Blende Semiconductors. *Phys. Rev. B* **1976**, *14* (2), 556. https://doi.org/10.1103/PhysRevB.14.556.

(18) Wang, C. S.; Klein, B. M. First-Principles Electronic Structure of Si, Ge, GaP, GaAs, ZnS, and ZnSe. I. Self-Consistent Energy Bands, Charge Densities, and Effective Masses. *Phys. Rev. B* **1981**, *24* (6), 3393. https://doi.org/10.1103/PhysRevB.24.3393.
